# LoRa Based IoT Platform for Remote Monitoring of Large-Scale Agriculture Farms in Chile

**DOI:** 10.3390/s22082824

**Published:** 2022-04-07

**Authors:** Mohamed A. Ahmed, Jose Luis Gallardo, Marcos D. Zuniga, Manuel A. Pedraza, Gonzalo Carvajal, Nicolás Jara, Rodrigo Carvajal

**Affiliations:** 1 Department of Electronics, Universidad Técnica Federico Santa María, Valparaíso 2390123, Chile; jose.gallardo.14@usm.cl (J.L.G.); manuelpedrazag@gmail.com (M.A.P.); gonzalo.carvajalb@usm.cl (G.C.); nicolas.jara@usm.cl (N.J.); 2 Escuela de Ingeniería Eléctrica, Pontificia Universidad Católica de Valparaíso, Valparaíso 2362804, Chile; rodrigo.carvajal@pucv.cl

**Keywords:** Internet of Things (IoT), smart farming, large-scale farms, remote monitoring, communication networks, LoRa technology

## Abstract

Nowadays, conventional agriculture farms lack high-level automated management due to the limited number of installed sensor nodes and measuring devices. Recent progress of the Internet of Things (IoT) technologies will play an essential role in future smart farming by enabling automated operations with minimum human intervention. The main objective of this work is to design and implement a flexible IoT-based platform for remote monitoring of agriculture farms of different scales, enabling continuous data collection from various IoT devices (sensors, actuators, meteorological masts, and drones). Such data will be available for end-users to improve decision-making and for training and validating advanced prediction algorithms. Unlike related works that concentrate on specific applications or evaluate technical aspects of specific layers of the IoT stack, this work considers a versatile approach and technical aspects at four layers: farm perception layer, sensors and actuators layer, communication layer, and application layer. The proposed solutions have been designed, implemented, and assessed for remote monitoring of plants, soil, and environmental conditions based on LoRaWAN technology. Results collected through both simulation and experimental validation show that the platform can be used to obtain valuable analytics of real-time monitoring that enable decisions and actions such as, for example, controlling the irrigation system or generating alarms. The contribution of this article relies on proposing a flexible hardware and software platform oriented on monitoring agriculture farms of different scales, based on LoRaWAN technology. Even though previous work can be found using similar technologies, they focus on specific applications or evaluate technical aspects of specific layers of the IoT stack.

## 1. Introduction

In Chile, agriculture is an important sector of development with a significant impact on economic growth. Chile is located in the southern hemisphere and has great opportunities for agriculture and forestry production due to the diversity in climates throughout the country and high standards for quality and safety [1]. Agriculture farms can be classified into small, medium, and large farms [2]. According to the 2007 national agriculture and livestock census, 73.4% of farms are small (less than 20 hectares), 19% medium size (20–100 hectares), and 7.6% are large farms (more than 100 hectares). For the future development of Chilean agriculture, the Ministry of Agriculture has developed a framework that targets four main axes: associativity, rural development, sustainability, and modernization of activity [1]. While agriculture plays an essential part in the economy of Latin American countries, technology is still far from being deployed in the field [3]. This research is directed toward the role of Internet of Things (IoT) technology in agriculture farms to modernize farm activity and improve management and efficiency.

Nowadays, modern technologies of sensors, communication networks, IoT, big data, and artificial intelligence are converging into complex cyber-physical systems and digital twins for different agriculture applications [4]. Agriculture applications include living plants/trees, agriculture products, agriculture fields/farms, agriculture buildings, and agriculture machines. These applications aim to enable stakeholders and farmers to remotely monitor, control and coordinate daily farm operations, and improve their decision making capabilities.

There are many studies and publications in the domain of IoT for smart agriculture that aim to solve different problems and provide various solutions [5,6,7,8,9,10,11,12,13,14,15,16,17,18]. The main issues for developing such solutions include hardware, networking, and platform challenges. The hardware challenges are related to hardware implementation and the harshness of the operational environments, including temperature, humidity, rain, dependence on a limited battery power source, and other dangers that may affect/destroy the electronic circuits. The networking challenges are related to the lack of communication infrastructure in rural areas, sensing large-scale farms, and connecting isolated areas. Wireless technologies will play an important role in deploying IoT-based solutions that enable sensor/actuator nodes to exchange data without human intervention. The platform challenges are related to building a suitable platform and applications that will allow real-time monitoring.

This work targets the networking challenges of large-scale farms in Chile to enable the integration of a massive number of sensor nodes, devices, and equipment. Long-range (LoRa) technology is a promising candidate to provide long-range communication over several kilometers based on low power consumption battery-powered devices. The main objective of this work is to design an IoT-based platform for remote monitoring of large-scale agriculture farms. The developed solution (hardware and software) can provide real-time tracking of labor activities for people and machines during harvest and measure different ambient and soil condition parameters for two fruit species, including blueberry and Hass avocado. The developed prototype offers a low-cost solution to access real-time data. The contribution of this article relies on proposing a flexible hardware and software platform oriented on monitoring agriculture farms of different scales, based on LoRaWAN technology. Even though previous work can be found using similar technologies, they focus on specific applications or evaluate technical aspects of specific layers of the IoT stack.

## 2. Related Work

Agriculture is one of the biggest food industries in the world and currently one of the most critical sectors of the economy in many countries due to the great demand for food required by the world population. As food demand increases every day, maximizing food production and minimizing food losses and costs are necessary. With the advancement of Internet of Things (IoT) technologies, more companies and organizations are interested in developing new solutions that enable remote monitoring and control to improve crop productivity. IoT is a promising technology that provides great opportunities to support many new applications and services in different domains such as smart home, smart city, smart industry, smart health, and smart agriculture [5,6,7,8,9].

The authors in [5] reviewed IoT applications in agro-industrial and environmental fields, identifying different application areas (monitoring, control, prediction, and logistics), trends, architectures, and open challenges covering the time period from 2006 to 2016. The authors in [6] present a comprehensive survey on the state-of-the-art for the role of IoT in agriculture, covering network architectures, technologies, and protocols. Furthermore, agriculture policies for the standardization of IoT-based agriculture and open research issues have been discussed. The authors in [7] developed a device named “FarmFox” to monitor soil health. The device uses REST API and TCP protocol. The developed sensor node monitors several soil parameters such as pH, turbidity, soil moisture, and temperature based on Wi-Fi technology and sends the sensor information to the end-user. The authors in [8] present a review of the applications of LoRaWAN for future smart farming, which include automatic irrigation systems, small/large arable farming, livestock and animal welfare, and greenhouse/indoor horticulture. The paper covers the basic applications of LoRaWAN for smart farms and highlights technology limits and future research directions. The author in [9] reviews various potential applications and challenges associated with IoT in agriculture and farming applications. Various case studies have been presented and explored regarding communication networks, cloud services, and hardware platforms. In [10], the author studies the impact of variant physical layer parameters on the performance of the LoRa network with respect to range, reliability, and RSSI in a tree farm located in Indiana, United States. The authors in [11] present a hardware and software system for the remote monitoring of vineyards. Two different nodes were developed and deployed to collect atmospheric data and soil parameters based on Wi-Fi technology in Ribeira Sacra, Spain. The authors in [12] propose a user-centered design model where each farmer decides their own installations. The experimental work considered a greenhouse with two levels of communication and processing nodes (edge and fog). In [13], the authors present the major applications of unmanned aerial vehicles (UAV) and IoT in smart farming. The work highlights connectivity requirements, network functionalities, and communication technologies for smart agriculture. In [14], the authors present an IoT scheme based on LoRa technology for long-range communication in the agriculture area. The monitoring parameters consist of humidity, temperature, soil pH and soil moisture. In [15], the authors present a comparative study for LPWAN such as Sigfox, LoRa, and NB-IoT for large-scale IoT deployment with respect to range, coverage, deployment, cost, battery life, latency, and scalability. The authors in [16] present a low-cost solution for an automatic irrigation system. The system consists of a LoRa network between sensor nodes and a local gateway with an internet connection through the Sigfox network. In [17], the authors present a case study for an IoT-based architecture for research and development in precision agriculture and ecological domains. The authors in [18] describe the AFarCloud project that aims to support the integration and cooperation of the agriculture system to offer better productivity, efficiency, food quality, and animal health. Table 1 shows comparisons between previous research work on IoT-based architectures for smart farming.

As connectivity is one of the most crucial factors that affect smart farming, there is a need for seamless connectivity and highly efficient communication among different devices and equipment [13]. Previous research has aimed to provide a sensor-specific solution such as soil health [7,11], atmospheric data [11], or technology-specific solutions such as Wi-Fi [11] and LoRa [10], where the detailed analysis of the developed system from different perspectives is missing. Furthermore, the selection of a particular technology for a specific application should be based on different requirements such as data rate, range, and power consumption. Low power wide area network (LPWAN) technologies will play an important role to support the deployment of different smart farming applications compared with traditional cellular systems [13]. For the adaption of precision agriculture services, the integration of heterogeneous IoT devices, the interoperability between different platforms and proprietary systems, and the cost of the required technological infrastructure are among the main barriers that hinder the adoption of new technologies [19]. Furthermore, different parameters should be taken into account for properly designing farming applications and services, such as coverage, cost, data delivery ratio, energy consumption, throughput, and collisions. The main contributions of this work are as follows:

- Propose an IoT-based platform for remote monitoring of large-scale agriculture farms. The proposed architecture consists of four layers: farm perception layer, sensors and actuators layer, communication network layer, and application layer.
- Develop a simulation model for the LoRa-based communication network of large-scale farms that enable remote monitoring of different sensor nodes and measurement devices in the field.
- Investigate the feasibility of using LoRa-based communication for large-scale farms concerning packet delivery ratio, number of collisions and throughput.
- Design, prototype, and implement a LoRa-based platform using low-cost devices that enable GPS LoRa tracking and real-time monitoring of ambient and soil conditions.

## 3. Materials and Methods

### 3.1. IoT-Based Architecture for Smart Farming

**Figure 1:**
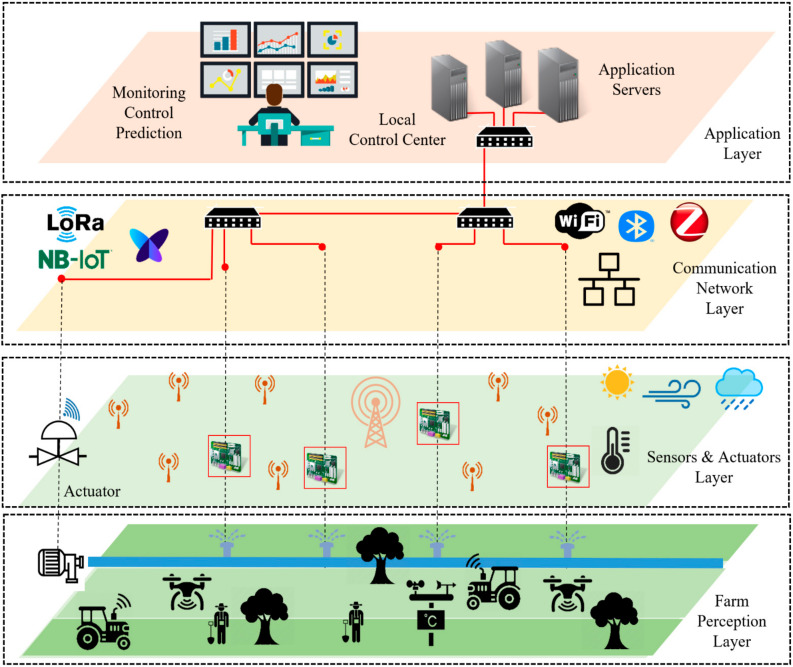
IoT-based architecture for smart farming.

This section presents the complete IoT-based architecture for smart farming [20,21]. The proposed architecture consists of four layers: farm perception layer, sensors and actuators layer, communication network layer, and application layer, as shown in Figure 1.

- *Farm Perception Layer*

This layer includes the different monitoring parameters such as weather conditions, soil moisture and humidity, plant monitoring, machine status, and water monitoring. The farm field includes various sensor nodes, actuators, and measurement devices. These nodes could be fixed sensor nodes, mobile sensor nodes, agro machines, etc. Sensor nodes are used for acquiring different measuring parameters covering, for example, plant, soil, and environment. The main objective of this layer is to enable data transmission with minimum human intervention. This work targets large-scale farms in Chile. The developed sensor nodes are designed to provide real-time tracking of labor activities (human and machines) during harvest for traceability and yield mapping. Furthermore, the system will be used for measuring the field conditions for two fruit species, including blueberry and Hass avocado.

- *Sensors and Actuators Layer*

Different sensor nodes and measurement devices are installed in the field to collect data (e.g., temperature sensors, soil sensors, weather stations, light sensors, humidity sensors, images, and videos). The collected data are transmitted to gateways or data collection points via wired/wireless communication. Monitoring data from this layer is used to keep track of the crop growth, where different parameters could be adjusted from the control center side, such as irrigation, fertilization, and pesticides.

- *Communication Network Layer*

The main function of the communication network layer is to enable data transmission from the farm perception layer to the application layer based on different long-range and short-range communication technologies. Such solutions include wired (Ethernet) and wireless (ZigBee, Bluetooth, Wi-Fi, 3G/4G, LoRa, NB-IoT, Sigfox) communication technologies that are used to exchange data with the help of field gateways distributed in the field. Other network equipment includes network switches, routers, gateways, and base station infrastructures.

- *Application Layer*

The application layer handles all the data received from sensor nodes and measuring devices via the communication network layer, as well as data storage, analytics, and visualization for different agriculture parameters such as weather conditions, irrigation information, soil quality, etc. The received data allows the end-user to remotely monitor and control farm operations. Thus, the application layer enables farm management, including planning and decision making.

### 3.2. LoRa-Based Architecture for Large-Scale Agriculture Farms

#### 3.2.1. LoRa Technology

LoRa technology is a promising candidate to support remote monitoring of large-scale agriculture farms due to their flexibility and simplicity. The LoRa network is based on two main components: LoRa physical layer and LoRaWAN. The proprietary LoRa physical layer was developed by the Semtech corporation based on spread spectrum modulation and chirp spread spectrum (CSS) [22], while the LoRaWAN is an open specification developed by LoRa Alliance [23]. Each LoRa transmission includes different parameters such as transmission power, spreading factors (SF), code rate (CR), center frequency, and bandwidth. These parameters affect the data rate, communication range and robustness of the signals [23,24].

- **Spreading Factor (SF)**: The ratio between the data symbol rate and chirp rate. The configuration of SF affects the data rate and coverage distance. The high spreading factor supports low data rates and a long communication range.
- **Code Rate (CR)**: Is the forward error correction rate that affects the airtime of packet transmission.
- **Central Frequency**: It depends on the industrial, scientific and medical (ISM) band used.
- **Bandwidth:** It affects the data rate of transmissions.

**Figure 2:**
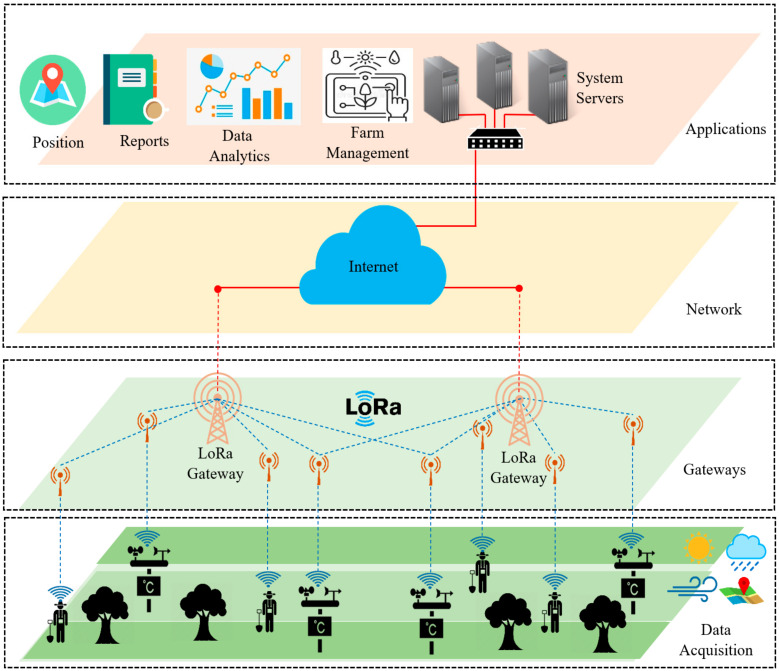
LoRa-based network architecture for smart farms.

In general, the communication network for a LoRa-based architecture consists of a hierarchical topology, formed by LoRa nodes which communicate through gateways. The architecture consists of LoRa nodes, LoRa gateways, network servers, and application servers. There is no association between LoRa nodes and the gateways; however, all gateways within the range of a LoRa node can receive messages. The central network server is responsible for processing the incoming packets, filtering the duplications, and forwarding the messages to the application servers. Figure 2 shows the main elements of the LoRa-based network architecture layout for smart farms.

- **Data Acquisition:** LoRa sensor nodes are responsible for collecting sensing data or control actions (actuators) through communication with gateways.
- **Gateways:** LoRa gateways are internet-connected devices responsible for receiving/transmitting data packets from/to different LoRa sensor/actuator nodes.
- **Network Server:** The network server is responsible for managing the LoRa gateways and handling the duplication of the received packets. Additionally, it is responsible for sending/scheduling data and acknowledgment to be transmitted for specific nodes.
- **Application Server:** The application server is responsible for handling LoRaWAN application layer, such as downlink/uplink data encoding/decoding and encryption/decryption.

#### 3.2.2. Design Requirements

This section provides the design requirements that should be considered while designing the IoT-based platform for large-scale farms. The performance evaluation metrics include modular design, unit cost, system scalability, robustness (ability to handle seasonal climate variability), and power management [5,6,19].

- **Hardware and energy efficiency requirements**: All sensor nodes and measuring devices in the farm perception layer are exposed to harsh environmental conditions such as temperature, humidity, rain, and wind that may affect the electronic circuits. These challenges should be considered during hardware design and implementation.
- **Cost requirements**: The total cost of the IoT system includes the price of sensor nodes, measuring devices, gateways, and base stations.
- **Scalability requirements**: The system back-end and databases should be able to support receiving information from a large number of sensor nodes and measuring devices. Additionally, the system should be able to add new hardware and/or software.
- **Network coverage requirements**: Wireless communication offers many advantages compared with wired-based solutions. However, network reliability, interference, and data loss should be evaluated as many sensor nodes use an unlicensed spectrum such as ZigBee, Wi-Fi, LoRa, etc.
- **Interoperability requirements**: Smart Agriculture farms should be able to integrate heterogeneous IoT devices, different proprietary systems, and/or platforms at different levels (data acquisition, communication network, and applications).

## 4. Results

### 4.1. LoRa Network Model and Simulation Results

**Figure 3:**
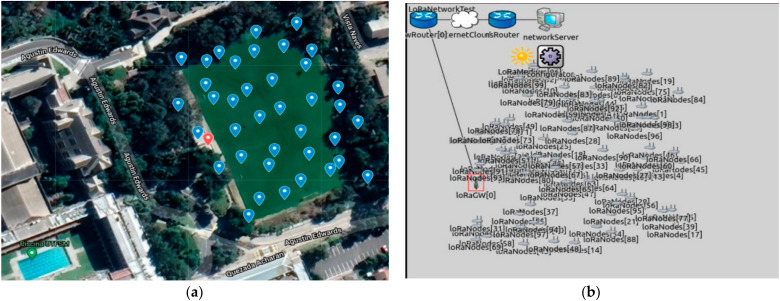
(**a**) UTFSM campus; (**b**) FLoRa simulation.

The LoRa communication network model is evaluated through simulation. The simulation models are based on the Open Source (Framework for LoRa) FLoRa Simulator, which is a simulation tool based on OMNeT++ [23,24,25]. FloRa Simulator includes different modules that simulate LoRa physical layer, LoRaWAN, gateways, and network servers. In FloRa, all the transmission parameters of the physical layer could be configured, such as the transmission power, code rate, bandwidth, center frequency, and spreading factor. Figure 3 shows the configured network topology for sensor nodes located in the playfield of the Universidad Técnica Federico Santa María (UTFSM) campus, Valparaiso, Chile.

This experiment aims to evaluate the performance of LoRa PHY and LoRaWAN technologies. The main metrics are data delivery ratio (DR), energy consumption (EC), throughput, number of collisions, and spreading factor (SF) distribution. The main features of LoRa links, energy consumer modules, and network elements are given below [20].

- **LoRa Links**: The LoRa transmission is successful if the received power is greater than the receiver sensitivity. The model is based on the log-distance path loss model and shadowing, where the path loss is based on the distance between the transmitter and receiver.
- **Energy Consumer Modules**: The three main states for the LoRa radio are transmit, receive, and sleep. The energy consumed is based on the amount of time spent by the LoRa radio in a particular state.
- **Network Elements:** The simulation model enables the network end-to-end simulation by modeling end-nodes, gateways, and network servers. The gateways and the network servers are communicating over IP such as Ethernet and Wi-Fi links.

The performance of the LoRa communication network is evaluated with adaptive data rate (ADR) and without ADR. Different configurations with different numbers of LoRa nodes (100, 200, 300, and 400) are considered. The main simulation parameters are given in Table 2. The simulation scenarios consider one LoRa Gateway connected to the network server.

#### 4.1.1. Delivery Ratio

**Figure 4:**
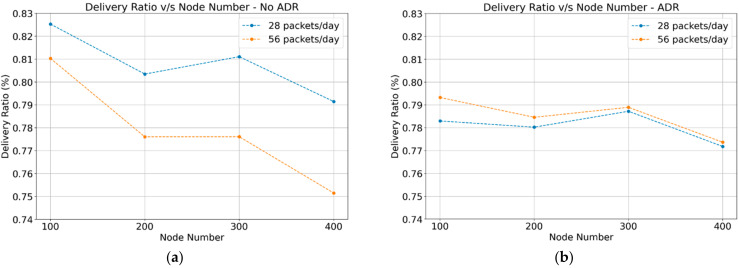
Delivery ratio: (**a**) ADR-disabled; (**b**) ADR-enabled.

The delivery ratio for LoRa networks is defined as the ratio between the number of messages correctly received by the network server and the total number of packets sent by every node. The delivery ratio gives a number between “0” and “1”. If the delivery ratio is close to “1”, then the network could be considered with an ideal behavior. However, a value close to “0” means that every transmitted packet may not reach the destination. Figure 4a,b show the results of the delivery ratio.

The ADR-enabled scheme is preferred for networks with a high density of nodes, as their performance does not decrease dramatically over time. For a small number of nodes, LoRa networks with ADR-disabled achieve a better delivery ratio than those with ADR-enabled due to their less saturated spectrum and fewer collisions. While increasing the number of nodes, the communication with ADR-disabled tends to be unstable compared with the ADR-enabled scheme, as this value decreases dramatically (about 0.75 in the case of 400 nodes).

#### 4.1.2. Energy Consumption

**Figure 5:**
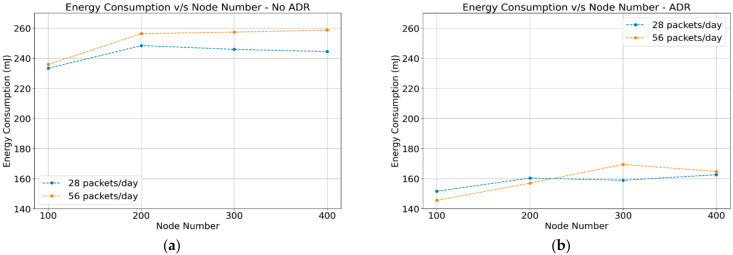
Energy consumption for end nodes: (**a**) ADR-disabled; (**b**) ADR-enabled.

The energy consumption for LoRa networks is defined as the accumulated energy consumption of every node in the network in millijoule (mJ). The ADR-disabled case shows similar results of power consumption for different packet frequency (28 and 56 packets) with a different number of sensor nodes (100, 200, 300, and 400). The energy consumption values did not show a significant difference in terms of the number of nodes in the network. Compared to the case with ADR-disabled, ADR-enabled communication schemes achieve better results with lower power consumption. Figure 5a,b show the results of the energy consumption for end nodes.

#### 4.1.3. Throughput

**Figure 6:**
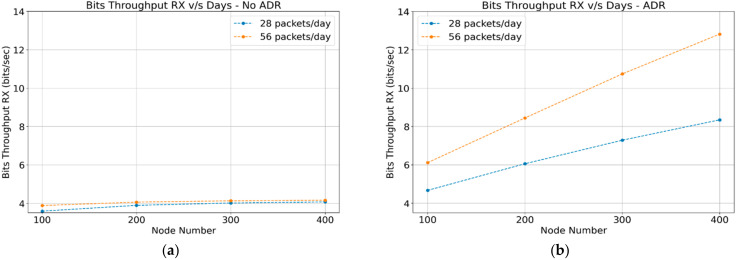
Gateway reception throughput: (**a**) ADR-disabled; (**b**) ADR-enabled.

**Figure 7:**
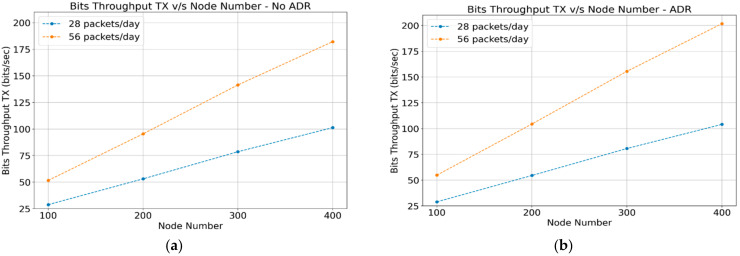
Gateway transmission throughput: (**a**) ADR-disabled; (**b**) ADR-enabled.

The reception throughput corresponds to all the acknowledgment and control messages perceived by the gateway, which are sent to the end devices, as shown in Figure 6a,b. The transmission throughput corresponds to all the data flow that the gateway perceives and is forwarded to the network server, which corresponds to the data packets transmitted by the end devices. Both scenarios (ADR-enabled and ADR-disabled) are very similar, as shown in Figure 7a,b. This confirms that networks operating with or without the “ADR” mechanism transmit the same amount of data.

#### 4.1.4. Collisions

**Figure 8:**
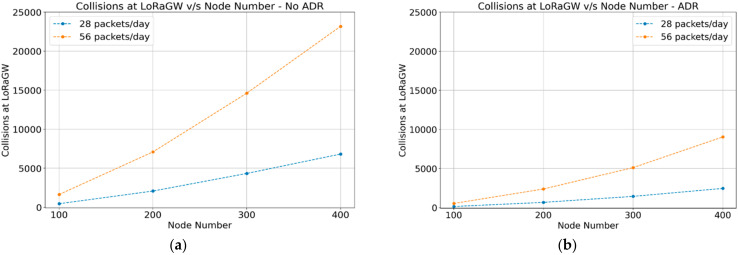
Collisions perceived by LoRa gateway (**a**) ADR-disabled; (**b**) ADR-enabled.

The collision metric is defined as the number of perceived collisions concerning the gateway. As shown in Figure 8a,b, there is a noticeable reduction in collisions in the case of ADR-enabled networks. The ADR-enabled mechanism helps in providing better energy usage, a higher reception throughput, and fewer collisions.

#### 4.1.5. SF Distribution

**Figure 9:**
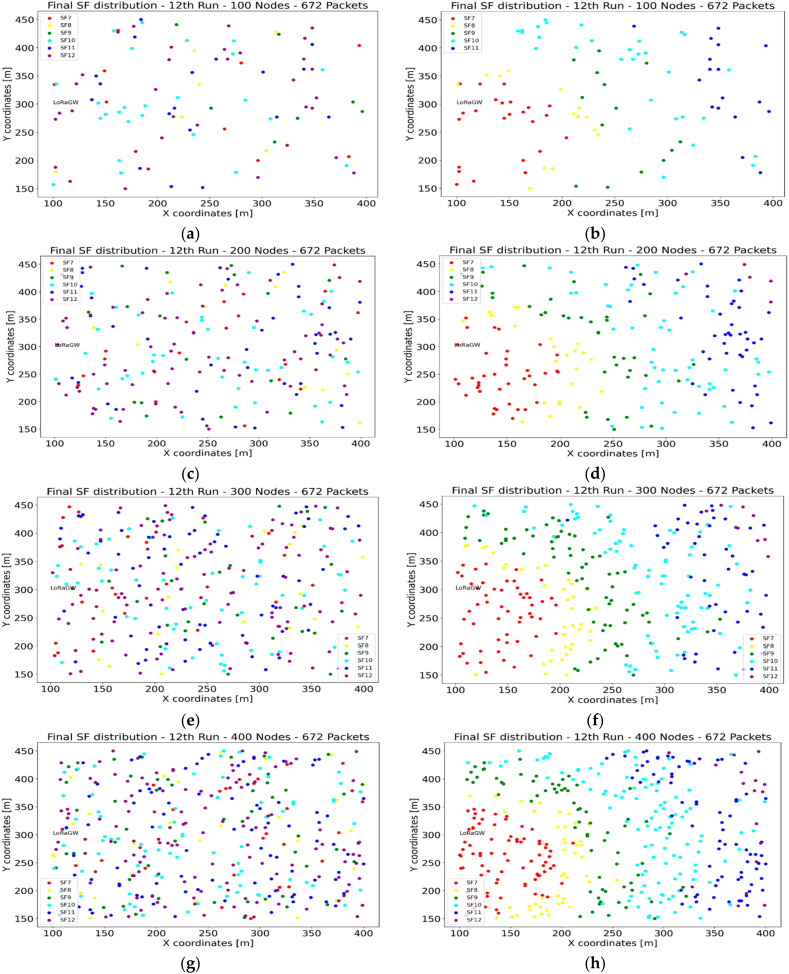
SF distribution with different number of sensor nodes: (**a**) 100 nodes ADR-disabled; (**b**) 100 nodes ADR-enabled; (**c**) 200 nodes ADR-disabled; (**d**) 200 nodes ADR-enabled; (**e**) 300 nodes ADR-disabled; (**f**) 300 nodes ADR-enabled; (**g**) 400 nodes ADR-disabled; (**h**) 400 nodes ADR-enabled.

This section analyzes and comments on the SF distribution, comparing the results with and without ADR. We chose the distribution result of the 29th run in each case, as it is one of the 30 executed runs. The chosen scenario is when nodes communicate with the network server every 15 min. When the ADR mechanism is disabled, no SF change is taken in the network, resulting in every node having its initial SF value. However, with ADR-enabled, at different communication ranges different SF values take place in order to provide better performance metrics, as shown in Figure 9.

#### 4.1.6. Discussion

The simulation results for data delivery show that more transmission packets imply a lower delivery ratio because more sensor nodes are transmitting simultaneously and occupying the same channel. In the case of ADR-enabled, a higher delivery ratio is achieved compared with ADR-disabled. We can conclude that ADR-enabled is recommended for networks supporting a high density of sensor nodes. At the same time, ADR-disabled offers better behavior for networks with a lower number of sensors and lower transmission frequency. As most of the sensor nodes in the agriculture field will be battery-powered, energy consumption is an essential factor for the network lifetime. The results of ADR-enabled showed a lower energy consumption than ADR-disabled. Concerning the throughput of transmission/reception at the gateway, the results showed that end devices are transmitting the same amount of data for ADR-enabled and ADR-disabled scenarios. However, the SF distribution in the case of ADR-enabled offers better performance and energy usage. In general, the network performance with ADR-enabled provides better performance for energy consumption, reception throughput, and collisions. A trade-off should be considered while designing a communication network with a small number of sensor nodes.

### 4.2. Localization Results

In large-scale farms, there are different activities that require information about locations, such as people and equipment [26]. This section aims to study different scenarios for localization using a low-cost solution, considering off-the-shelf GPS LoRa nodes, in order to evaluate the accuracy and operation capability in a real environment. The collected real data from the GPS nodes are compared with different high precision and high-cost mobile devices.

In this work, the GPS LoRa tracking nodes are called “Farm Tracker”. These Farm Trackers can be used by persons/machines during the cultivation process to acquire GPS data for real-time tracking and harvest management and relay it to the control center using LoRa communication. The Farm Tracker system has been implemented and tested in UTFSM (Universidad Técnica Federico Santa María) campus, Valparaiso, Chile. The collected data from the GPS LoRa nodes are compared with Geo Tracker Mobile applications installed on different high precision and high-cost mobile devices. The current prototype is a proof-of-concept and can be used in various farm applications.

**Figure 10:**
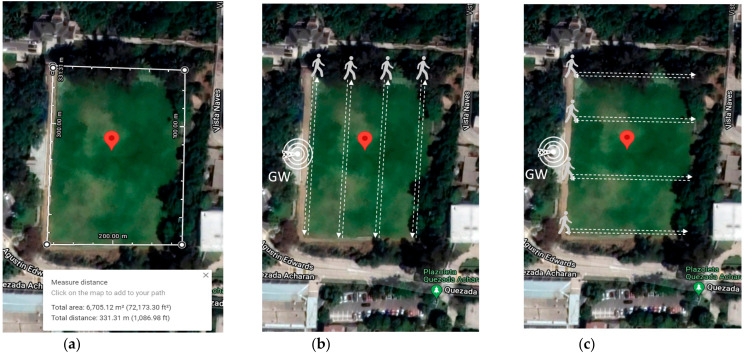
Experiment scenarios for Farm Tracker (**a**) The playfield of Universidad Técnica Federico Santa María, Valparaiso, Chile (Image from Google Earth); (**b**) Scenario 1 movement direction and route; (**c**) Scenario 2 movement direction and route. GW: LoRa Gateway.

Figure 10a shows the satellite map for the experimental scenarios for the Farm Trackers. The GPS LoRa nodes setup is implemented and tested on the playfield located around the coordinates 33°02′05.3″ S 71°35′38.5″ W of the UTFSM campus. The total area is about 6705 m^2^. The playfield is covered with grass and surrounded by trees and buildings. There are no obstacles (e.g., buildings, trees) in the intermediate terrain. The location of the LoRa gateway (Dragino DLOS8) is shown in Figure 10b,c. This is an outdoor LoRaWAN gateway which includes a 1 × SX1301 + 2 × 1257 LoRa transceiver. Table 3 shows the technical specifications for the Dragino DLOS8 LoRa Gateway [27].

The experiments were carried out on 26 January 2022 at 05:00 PM. The Geo Tracker Mobile application was the software installed on four mobile phone devices (D1–D4) to collect data during the experiment. A smartwatch (D1) was used for data collection among the devices. For scenario 1, the closest and longest distance between the mobile devices and the LoRa Gateway is about 50 m and 90 m, respectively. For scenario 2, the closest and longest distance between the mobile devices and the LoRa Gateway is about 20 m and 50 m, respectively. Table 4 shows the mobile device types, brands, and models. The data collected from the field are stored in a laptop connected to the LoRa gateway for further analysis. The results of the GPS LoRa nodes are visualized using OpenStreetMap.

**Figure 11:**
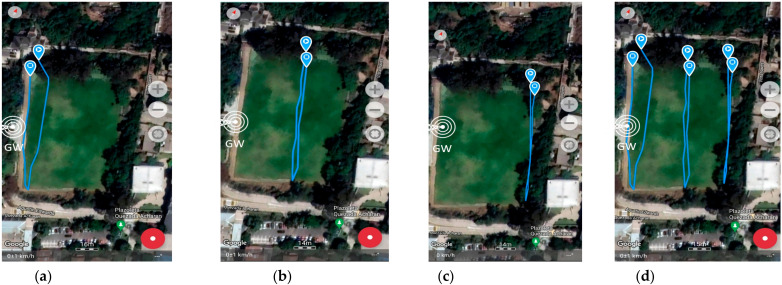
GPS LoRa results in experiment site for scenario 1 (movement direction and route using Geo Tracker Mobile APP) (**a**) Smart Watch (**b**) LG model K41S (**c**) Samsung model S20 (**d**) All data. GW: LoRa Gateway.

**Figure 12:**
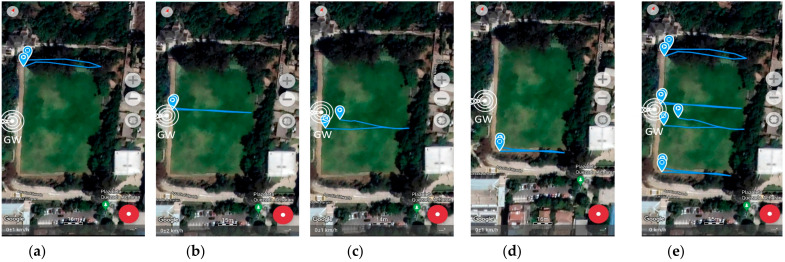
GPS LoRa results in experiment site for scenario 2 (movement direction and route using Geo Tracker Mobile APP) (**a**) Smart Watch (**b**) LG model K41S (**c**) Samsung model S20 (**d**) Xiaomi model POCO F3 (**e**) All data. GW: LoRa Gateway.

The measurement was collected using GPS LoRa nodes (ESP32 TTGO GPS NEO6 board). In the backside of the module, there is a battery holder for a Li-Ion battery which enables the portable operation of the nodes. The GPS LoRa nodes are named: ESP32-1, ESP32-2, ESP32-3, ESP32-4, and ESP32-5. The node ESP32-2 had a problem during the experiment and was not considered for data collection. Smart mobile devices used are smartwatch (D1), LG K41S (D2), Samsung S20 (D3), and Xiaomi POCO F3 (D3). The measurements were collected by four people. Each person ported a GPS LoRa node (ESP32 TTGO GPS NEO6) and a mobile device with Geo Tracker Mobile APP. The four persons start moving simultaneously and in the same direction. Table 5 shows the parameter settings for the GPS LoRa nodes. The measurements were collected using four LoRa nodes (ESP32-1, ESP32-3, ESP32-4 and ESP32-5). Figure 11 and Figure 12 show the movement direction and routes for scenario 1 and scenario 2, respectively, using the Geo Tracker Mobile APP.

**Figure 13:**
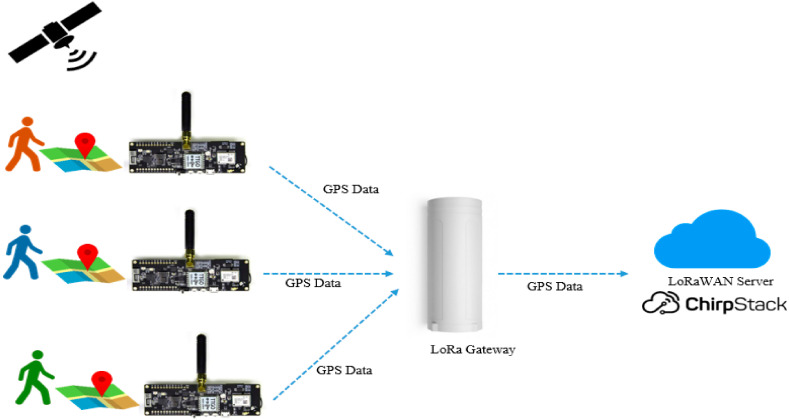
Schematic diagram for the Farm Tracker network.

As explained in the previous section, the prototype of the GPS LoRa nodes consists of four nodes. Each node includes a LoRa transceiver, GPS receiver, a microcontroller, and Li-Ion battery. Figure 13 shows the schematic diagram for the Farm Tracker network. The network is configured as a star topology where GPS LoRa nodes transmit data to a central system using LoRa communication. For each LoRa frame, the payload includes the device ID and GPS data information. The received data can show, in real-time, the locations of the moving nodes and visualize them on a map. In this work, the system has been designed to determine the real-time locations of farmworkers inside the farm during different cultivation activities. However, the system can be extended to support other tracking applications.

**Figure 14:**
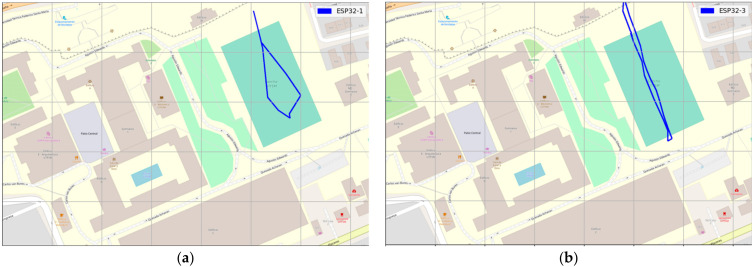

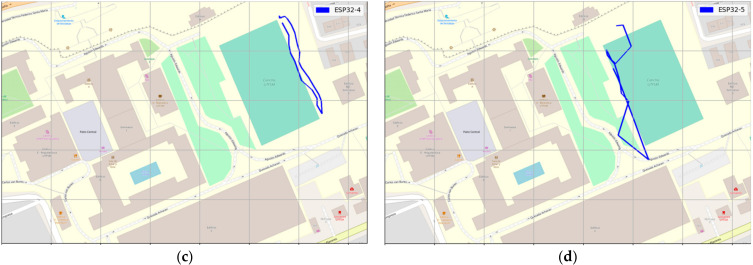
GPS LoRa results for Scenario 1 movement direction and route using LoRa GPS Nodes (**a**) ESP32-1 (**b**) ESP32-3 (**c**) ESP32-4 (**d**) ESP32-5.

**Figure 15:**
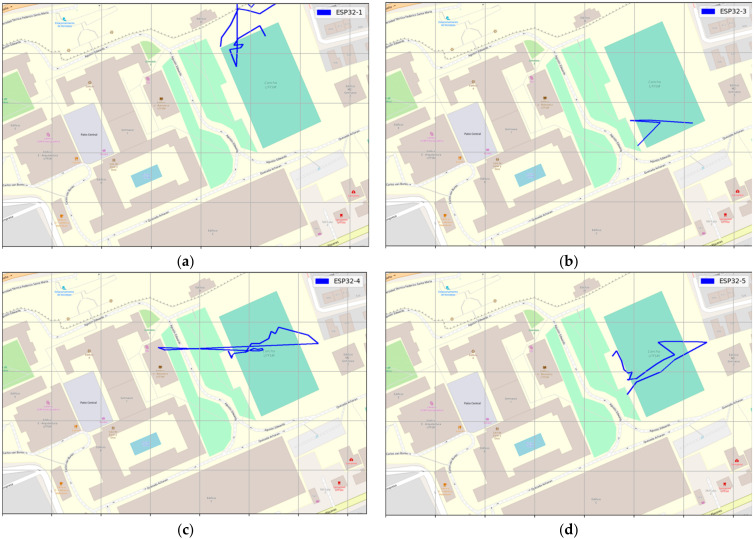
GPS results for Scenario 2 movement direction and route using LoRa GPS Nodes (**a**) ESP32-1 (**b**) ESP32-3 (**c**) ESP32-4 (**d**) ESP32-5.

**Figure 16:**
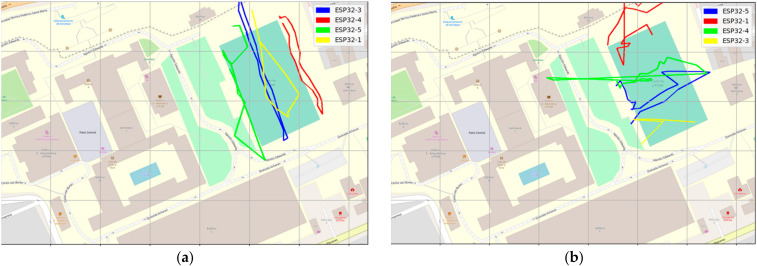
Aggregate GPS results for Scenario 1 movement direction and route using LoRa GPS nodes (**a**) Scenario 1 movement direction and route (**b**) Scenario 2 movement direction and route.

At this stage, the most critical metric was to test the ability to receive GPS LoRa data from different nodes and GPS accuracy. Figure 14, Figure 15 and Figure 16 show the results of scenario 1 and scenario 2, respectively, for the movement direction and route using GPS LoRa nodes. The collected data includes the information of Device ID, GPS location (latitude and longitude), and a time stamp. The results verify the success of the GPS data reception. Figure 16 shows aggregated GPS LoRa results for movement direction and route of scenario 1 and scenario 2. In large-scale farms, such information is valuable for harvest management [26] and human/machine safety [28], which could be used for improving the field logistics and creating yield maps.

## 5. Implementation

**Figure 17:**
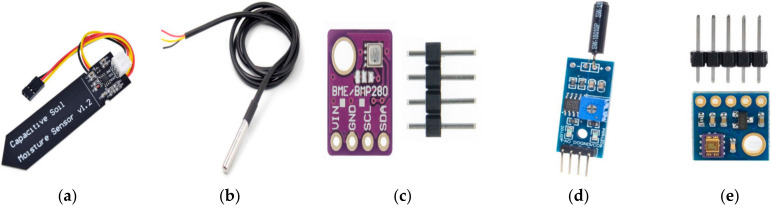
Real sensor nodes used for the design (**a**) Capacitive Soil Moisture; (**b**) DS18B20; (**c**) BME280; (**d**) SW-18010-P; (**e**) GYML8511.

This section explains the design and implementation of the low-cost sensor nodes and data transmission. Five sensor nodes have been designed and implemented for monitoring different parameters of ambient and soil conditions, including temperature, humidity, moisture, etc. Table 6 shows the main specifications of sensors used in the design. There are two types of measurements: ambient parameters (temperature, pressure, relative humidity, vibration, and ultraviolet) and soil parameters (soil moisture and temperature). The complete list of the main sensors is shown in Figure 17.

**Figure 18:**
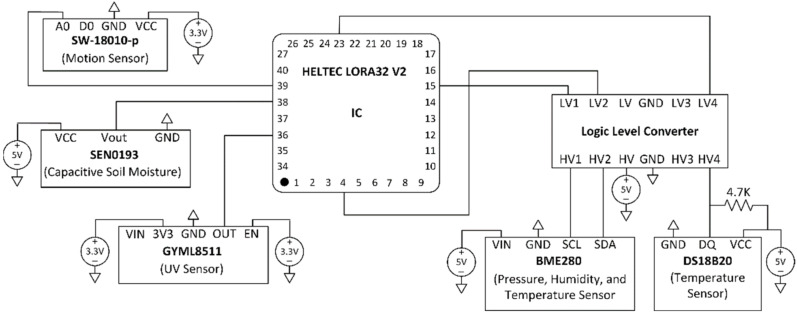
Schematic diagram of the node circuit.

**Figure 19:**
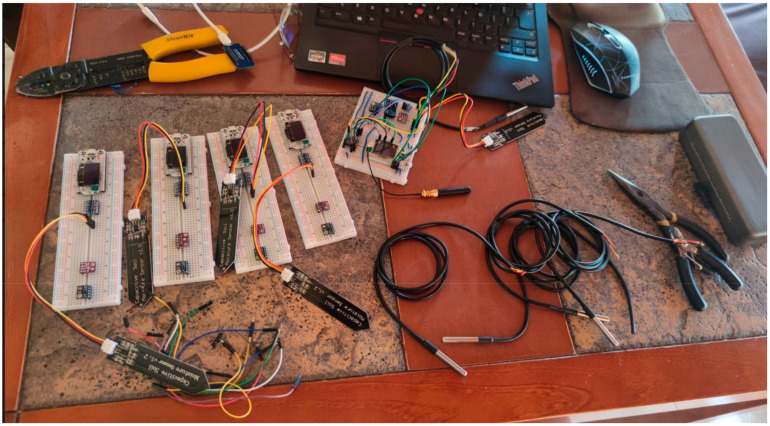
Assembling of the sensor nodes.

**Figure 20:**
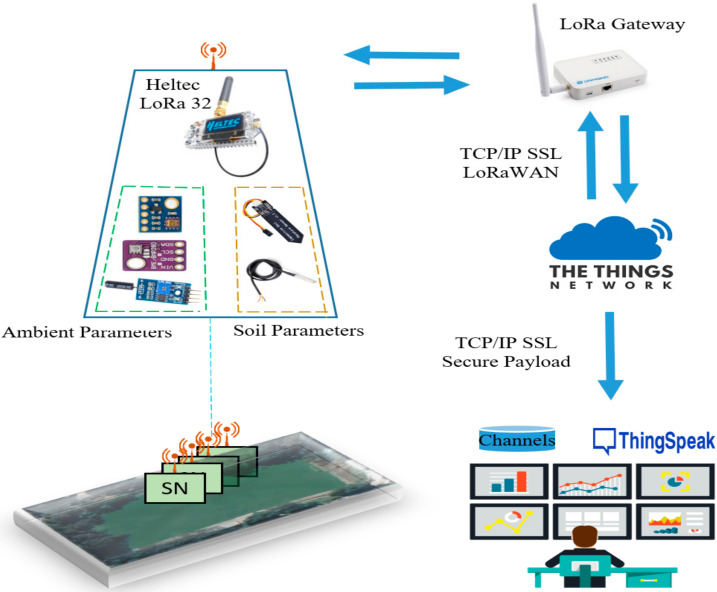
System architecture.

**Figure 21:**
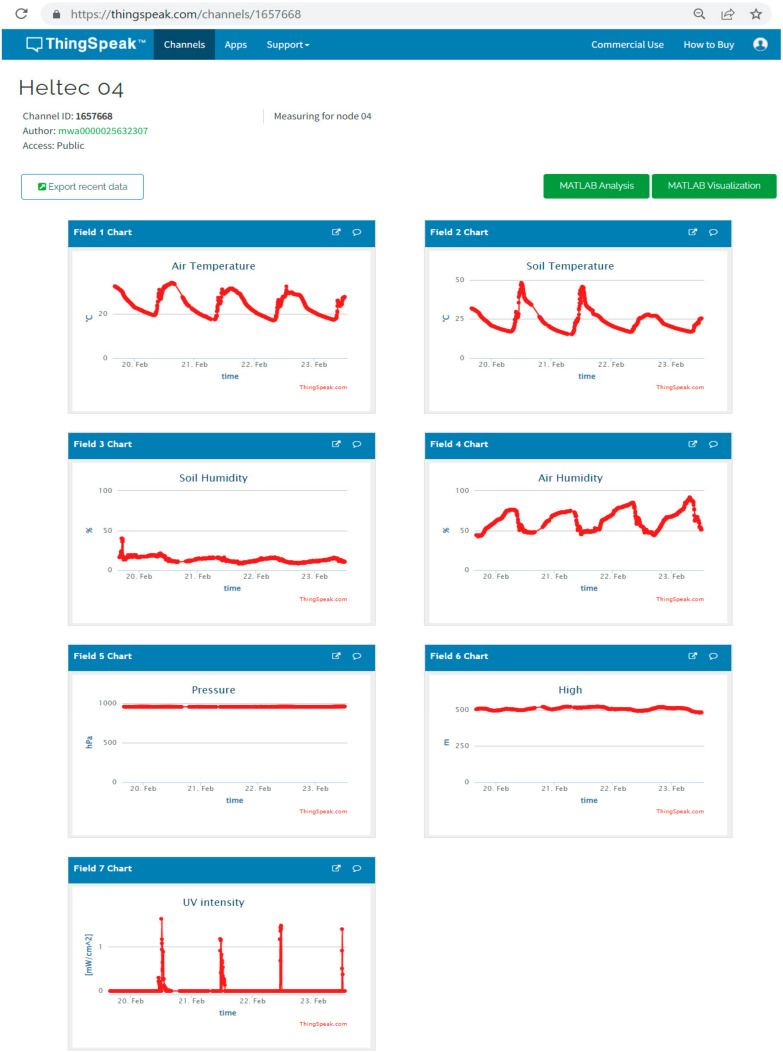
Dashboard for sensor values obtained from sensor node “Heltec 04” during the period from 19–23 February 2022. Data are available online using the following link (https://thingspeak.com/channels/1657668).

We have implemented five sensor nodes as proof of concept at this stage. The nodes have been installed in an indoor environment to collect different monitoring parameters. Figure 18 shows the schematic diagram of the node circuit, while Figure 19 shows the assembling of the sensor nodes. Figure 20 shows the complete system architecture where the configuration of LoRaWAN server on The Things Network (TTN) stack has been configured to receive monitoring data from LoRa sensor nodes and visualized using ThingSpeak Platform. Figure 21 shows the measurements from sensor node number “Heltec 04” since 19 February 2022. The data are available online using the following links: Heltec 04 (https://thingspeak.com/channels/1657668), Heltec 03 (/channels/1657672), Heltec 02 (/channels/1657658), Heltec 01 (/channels/1657655), and Heltec 00 (/channels/1657654). The stored data on ThingSpeak platform includes air temperature, air humidity, soil temperature, soil humidity, pressure, and UV intensity, as shown in Figure 21.

## 6. Discussion

The applications of IoT and LoRa technologies have received great interest for their support of different applications in large-scale agriculture farms. This work presented the results of the hardware and software platform which target large-scale farms. The proposed platform overcomes the challenge of legacy solutions, including hardware and services, to support extended coverage. Detailed descriptions for the proposed architecture have been given, including the communication between sensor nodes, LoRa gateways, and the network server for real applications in Chile.

Although most of the research has been carried out by conducting different experiments for different crop farms, this work has focused on designing and building the complete sensor network with low costs (infrastructure investment and operating expenses). Furthermore, the developed LoRa simulation model using FloRa showed the network performance with respect to data delivery ratio, energy consumption, throughput, number of collisions and spreading factor distribution.

The main results include:

- Propose IoT-based architecture consisting of four layers: farm perception layer, sensors and actuators layer, communication network layer, and application layer.
- Present a complete system architecture, components, interconnections, and detailed implementation of the proposed solution.
- Design, prototype and implement LoRa-based platform using low-cost devices that enable GPS LoRa tracking (Farm Tracker) and remote real-time monitoring of ambient and soil conditions.
- All the components have been acquired and purchased from the Chilean market.
- The platform allows the integration of new sensor nodes as well as data visualization.
- The system could be adjusted to collect data locally in a local database without the need to access the internet.
- The current cost of the sensor node is 53,500 CLP.
- The ongoing research work aims to:
- Develop the energy harvest module using a small solar panel to support the autonomous operation of the developed sensor nodes for remote real-time monitoring of ambient and soil conditions.
- Add more low-cost sensors to the current prototype, such as wind speed and wind direction.
- Enable notification for critical situations and abnormal values (sudden change from the rated values) as well as advanced data processing techniques. Such information will help to improve farm operation and productivity.
- Integrate the monitoring data collected from moving nodes such as drones and unmanned ground vehicles (UGV).

## 7. Conclusions

This work developed an innovative LoRa-based platform that provides an inexpensive solution for real-time monitoring of different applications in large-scale agriculture farms, including GPS LoRa tracking and monitoring atmospheric/soil data. The proposed IoT architecture consisted of four layers: farm perception layer, sensors and actuators layer, communication network layer, and application layer. The data transmission in the proposed solution is based on low power wide area network (LPWAN) using LoRaWAN, which meets the design requirements of large-scale agriculture farms. The system has been validated using an actual prototype as well as simulation. Data from different sensor nodes are transmitted to the LoRAWAN Gateways and then to The Things Network (TTN) platform. The TTN platform is responsible for data collection, formatting, and rerouting to ThingSpeak platform for data storage, visualization, and analysis. The received data opens new opportunities for different data analytics solutions which enable real-time data processing and support various forecasting services for farm management. Our ongoing work is to develop actuator nodes for controlling and automating different farm operations and processes such as irrigation. The developed sensor/actuator nodes will be installed in different real environments (mountains and rural areas). Although this work was focused on large-scale agriculture farms, it could be applied for other applications with similar requirements in the smart grid and smart cities domain.

## Figures and Tables

**Table 1:** Comparison among previous research work on IoT-based architectures for different smart farming applications.

Ref.	Type	Perception Layer	Network Layer	Application Layer	Contribution
[5]	Survey	Yes	Yes	Yes	The paper reviewed different IoT applications in agro-industrial and environmental fields, identifying the application areas (monitoring, control, prediction, and logistics), trends, architectures, and open challenges, covering the time period from 2006 to 2016.
[6]	Survey	Yes	Yes	Yes	The paper presented a comprehensive survey on the state-of-the-art for the role of IoT in agriculture, covering network architectures, technologies, and protocols. Furthermore, the work discussed the agriculture policies for the standardization of IoT-based agriculture and open research issues had been discussed.
[7]	Technical	Yes	Wi-Fi	Yes	The paper developed a device named “FarmFox” to monitor soil health. The device uses REST API and TCP protocol. Based on Wi-Fi technology, different monitoring parameters were collected for soil conditions such as pH, turbidity, soil moisture, and temperature.
[8]	Survey	Yes	LoRaWAN	Yes	The paper presented the applications of LoRaWAN for future smart farming. The paper covered the basic applications of LoRaWAN for smart farms and highlighted the technology limits.
[9]	Survey	Yes	Yes	Yes	The paper reviewed various potential applications and challenges associated with IoT in agriculture and farming applications. Various case studies were presented and explored regarding communication networks, cloud services, and hardware platforms.
[10]	Technical	Yes	LoRaWAN	Yes	The paper studied the impact of the physical layer parameters on the performance of the LoRa network with respect to range, reliability, and RSSI in a tree farm located in Indiana, USA.
[11]	Technical	Yes	Wi-Fi	Yes	The paper presented a hardware and software system for remote monitoring of vineyards. Two different nodes were developed and deployed to collect atmospheric data and soil parameters in Ribeira Sacra, Spain, based on Wi-Fi technology.
[12]	Technical	Yes	Wi-Fi	Yes	The paper proposed a user centered design model where each farmer decides their own installations. The experimental work considered a greenhouse with two levels of communication and processing nodes (edge and fog).
[13]	Survey	Yes	Yes	Yes	The paper presented the major applications of IoT and unmanned aerial vehicles (UAV) in smart farming, highlighting connectivity requirements, network functionalities, and communication technologies.
[14]	Technical	Yes	LoRaWAN	No	The paper presented an IoT scheme based on LoRa technology for long-range communication in the agriculture area. The monitoring parameters are humidity, temperature, soil pH, and soil moisture.
[15]	Survey	No	Sigfox LoRaWANNB-IoT	No	The paper presented a comparative study for LPWAN technologies such as Sigfox, LoRa, and NB-IoT with respect to range, coverage, deployment, cost, battery life, latency, and scalability.
[16]	Technical	Yes	LoRaWAN	Yes	The paper presented a low-cost solution for an automatic irrigation system. The system consists of a LoRaWAN network between sensor nodes and a local gateway with an internet connection through the Sigfox network.
[17]	Technical	Yes	LoRaWAN	Yes	The paper describes the AFarCloud project that aims to support the integration and cooperation of the agriculture system to offer better productivity, efficiency, food quality, and animal health.
[18]	Technical	Yes	3G	Yes	The paper presented a case study for a private IoT-based architecture aimed at the use of research and development for precision agriculture and ecological domains.
Present Work	Technical	Yes	LoRaWAN	Yes	The paper developed a hardware and software platform for remote monitoring of large-scale agriculture farms based on LoRaWAN technology. Different nodes have been developed and deployed to collect atmospheric data, soil parameters and GPS locations in Universidad Técnica Federico Santa María, Valparaiso, Chile.

**Table 2:** Simulation parameters for FloRa Simulator.

Parameter	Value
Carrier Frequency	915 MHz
Bandwidth	125 kHz
Coding Rate	4/8
Spreading Factor	7 up to 12
Transmission Power	2 dBm up to 14 dBm
Path Loss	3.57 dB
Path Loss Distance	40 m
Path Loss Exponent	2
Number of Gateways	1
Number of Nodes	100, 200, 300, 400
Payload Size (end-devices)	20 Bytes
Payload Size (control)	15 Bytes
Packets sent	{28, 56} per day

**Table 3:** Technical specifications for Dragino DLOS8 LoRa gateway.

Name	Specifications
Processor	400 MHz AR9331, 64 MB RAM, 16 MB Flash
Interfaces	10 M/100 M RJ45 Ports x 2 Wi-Fi 802.11 b,g,n LoRaWAN Wireless
LoRa Interface	1 × SX1301 + 2 × 1257
Power supply	12 V DC

**Table 4:** Mobile devices characteristics. GNSS: Global Navigation Satellite System.

Device Number	Device Brand	Device Model	GNSS
Device 1 (D1)	SMARTWATCH	AMAZFIT PACE	GPS, GLONASS
Device 2 (D2)	LG	K41S	GPS, A-GPS, GLONASS, BeiDou
Device 3 (D3)	SAMSUNG	S20	GPS, A-GPS, GLONASS, BeiDou, Galileo
Device 4 (D4)	XIAOMI	POCO F3	A-GPS, GLONASS, BeiDou, Galileo, QZSS, NavIC

**Table 5:** Setting parameters for the GPS LoRa nodes.

Parameter	Value	Parameter	Value
Carrier Frequency	915 MHz	Spreading Factor	7
Bandwidth	125 kHz	Number of Gateways	1
Coding Rate	4/5	Number of Nodes	4

**Table 6:** Main characteristics of sensors used for measuring soil and ambient conditions.

Target	Sensor Name/Number	Sensor Type	Specifications
Soil Parameters	Capacitive Soil Moisture v1.2 *	Soil moisture	3-Pin Sensor interface, Analog output, Operating voltage: 3.3–5.5 V
	DS18B20 ***	Temperature	Range: −55 to 125 °C, Accuracy: ±0.5 °C from −10 °C to +85 °C, Operating voltage: 3.3–5.5 V
Ambient Parameters	BME280 **	Temperature Pressure Relative Humidity	Range: −40 °C to 85 °C, Resolution: 0.01 °C, Accuracy: 1 °C Range: 300 to 1100 hPa, Resolution: 0.16 Pa, Absolute Accuracy: 1 hPa Range: 0–100% RH, Accuracy: ±3%
	SW-18010-P ****	Vibration	4-Pin Sensor interface, Digital output, Operating voltage: 3.3–5 V
	GYML8511 *****	Ultraviolet	Detection wavelength band 280 nm to 390 nm, Analog output, Operating voltage: 3–5 V

* https://maxelectronica.cl/temperatura-y-humedad/519-sensor-capacitivo-de-humedad-de-suelo-v12.html (accessed on 10 January 2021); ** https://maxelectronica.cl/temperatura-y-humedad/13-sensor-digital-de-temperatura-ds18b20-1-wire-impermeable.html (accessed on 10 January 2021); *** https://afel.cl/producto/sensor-barometrico-bme280/ (accessed on 10 January 2021); **** https://depaquete.cl/index.php?route=product/product&product_id=112&search=SW-18010-P (accessed on 10 January 2021); ***** https://afel.cl/producto/sensor-ultravioleta-uv-gyml8511/ (accessed on 10 January 2021).

## Data Availability

Not applicable.

## Nomenclature

IoT	Internet of Things
REST	Representational State Transfer
API	Application Programming Interface
TCP	Transmission Control Protocol
LoRaWAN	LoRa Wide Area Network
RSSI	Received Signal Strength Indicator
UAV	Unmanned Aerial Vehicle
LoRa	Long Range
LPWAN	Low Power Wide Area Network
NB-IoT	Narrowband-IoT
GPS	Global Position System
DR	Delivery Ratio
EC	Energy Consumption
SF	Spreading Factor
ADR	Adaptive Data Rate
CSS	Chirp Spread Spectrum
TTN	The Things Network
SNR	Signal to Noise Ratio
